# A multiscale assessment of bowel impairment in an Italian multiple sclerosis cohort

**DOI:** 10.1038/s41598-023-48317-9

**Published:** 2023-12-11

**Authors:** Eleonora Tavazzi, Antonio Zito, Cristina Montomoli, Niels Bergsland, Elena Colombo, Alessandro La Malfa, Roberto Bergamaschi

**Affiliations:** 1 IRCCS Fondazione Istituto Neurologico C.Mondino, Via Mondino 2, 27100 Pavia, Italy; 2 Unit of Biostatistics and Clinical Epidemiology, Department of Public Health, Experimental and Forensic Medicine, University of Pavia, Pavia, Italy; 3 Department of Neurology, School of Medicine and Biomedical Sciences, Buffalo Neuroimaging Analysis Center, University at Buffalo, State University of New York, Buffalo, NY USA

**Keywords:** Neuroscience, Neurology

## Abstract

Bowel dysfunctions (BD) in multiple sclerosis (MS) are under reported despite their clinical relevance. Scales usually applied do not thoroughly assess constipation and fecal incontinence. Instead, a proper qualitative and quantitative description of these symptoms might have relevant clinical and scientific consequences. The aim of this project is to study the prevalence of BD in a cohort of persons with MS (pwMS). Four-hundred and forty-seven pwMS (330 relapsing–remitting MS-RRMS and 117 progressive MS-PMS) were recruited. Three different questionnaires were administered: the neurogenic bowel dysfunction score (NBDS), the Wexner constipation scale (WexCon) and the Wexner incontinence scale (WexInc). All the scales were divided in subscores according to symptom severity. The prevalence of BD, considered as NBDS > 0, was 53.7%. Mean scores in pwMS group were as follows: NBDS 2.6 (SD 3.5), WexInc 1.1 (SD 2.4), WexCon 4.4 (SD 5.9). NBDS, WexCon and WexInc were significantly higher in PMS vs RRMS (p < 0.001), and significantly associated with disease duration, EDSS, multiple sclerosis severity score (p < 0.001), as well as with each other (p < 0.001). Our study confirms the presence of bowel dysfunctions in a large group of pwMS with a wide range of disability and their association with progressive disease phenotype and clinical disability.

## Introduction

Multiple sclerosis (MS) is a chronic neurological disease affecting the central nervous system (CNS) and resulting in a multi-functional impairment, with various possible symptoms ranging from motor deficits to visual or sensory disturbances. Among others, sphincter dysfunctions leading to bowel/bladder impairment are of particular interest for a number of reasons. They have an impact on quality of life, mainly on social and work-related activities, and are listed among the factors affecting the employment status of people with MS (pwMS)^1–3^. Despite their negative role on the well-being of pwMS, sphincter-related symptoms have typically been inadequately investigated by clinicians and are under-reported by patients, leading to an underestimation of their actual prevalence^4^. The strong association between bowel dysfunctions and physical and mental health as well as with the level of satisfaction with life situation^2^, fatigue^5^ and cognitive disability further underline the need to carefully assess this aspect in pwMS^6^. The relevance of sphincter dysfunctions in MS is related also to socio-economic factors: significantly higher costs for medicines for constipation in MS as well as inpatient costs related to urinary tract infections, which are often an indirect consequence of bowel dysfunction^7^.

Bowel impairment encompasses different symptoms, from constipation to incontinence, with an estimated prevalence between 39 and 73%^8^. The reasons of this wide range might be attributable to the different methods used for its assessment as well as the clinical and demographic data of selected patients. One of the most commonly applied tools to investigate the presence and quality of bowel dysfunction is the Neurogenic Bowel Dysfunction Score (NBDS)^9^. Despite being simple and quick to complete, a main limitation of this tool resides in the fact that it does not thoroughly investigate constipation and incontinence.

Against this background, the current study aimed at assessing the prevalence of bowel dysfunction in a large cohort of pwMS, applying different scales, to separately define the prevalence and severity of both bowel constipation and fecal incontinence.

## Materials and methods

MS outpatients were consecutively recruited in this cross-sectional study between December 2020 and December 2021 at IRCCS Mondino Foundation, Pavia, Italy. Inclusion criteria were: 1. diagnosis of clinically definite MS according to the 2017 McDonald criteria, 2. age > 18 years old. The only exclusion criterion was the impossibility to respond properly to the questionnaires, due to severe cognitive impairment.

Demographic and clinical data were collected for each patient (age, sex, disease duration, level of disability quantified by means of Expanded Disability Status Scale (EDSS)); Multiple Sclerosis Severity Score was derived by disease duration and EDSS, using the scale provided by Roxburgh et al.^10^ Considering the small number of patients with primary progressive MS (PPMS), they were merged with those with secondary progressive MS (SPMS) into a single group of patients with progressive MS (PMS).

Bowel dysfunction was investigated using 3 different questionnaires: NBDS, Wexner Incontinence scale and Wexner constipation scale. The study was approved by the local Ethics Committee of the IRCCS Policlinico S. Matteo and conducted accordingly to the Declaration of Helsinki. Patients signed an informed consent upon study recruitment.

### NBDS

NBDS is a self-administered multiple-choice questionnaire composed of 10 items investigating several aspects related to bowel dysfunctions, such as the frequency of bowel movements headache/perspiration/discomfort during defecation, use of medication for constipation or fecal incontinence, time spent defecating, digital stimulation or evacuation, frequency of fecal incontinence, flatus, and perianal skin problems. The global score ranges from 0 to 47, and the severity of bowel impairment can be assessed by categorizing the final score into subgroups as follows: normal (0); very minor (1–6); minor (7–9); moderate (10–13); severe (≥ 14)^11^.

### Wexner incontinence

The Wexner Incontinence (WexInc) scale comprises 5 multi-choice items, ranging from 0 (never) to 4 (daily), assessing the type of incontinence (solid/liquid stool), the presence of gas, lifestyle changes and the need to wear a pad^12^. Despite its simplicity, it is thorough in assessing the most important features of fecal incontinence and is the most widely used, and cited, fecal incontinence scale^13^. In the current study, WexInc was divided into severity subgroups according to cut-off scores as follows: normal (0); mild (1–5); moderate (6–10); severe (> 10).

### Wexner constipation

The Wexner Constipation (WexCon) scoring system ranges from 0 to 32 and evaluates 8 factors such as the frequency of bowel movements, painful evacuation effort, incomplete evacuation, abdominal pain, minutes needed for any attempt, assistance for evacuation, unsuccessful evacuation attempts per 24 h, and duration of constipation. The WexCon showed a good correlation with objective instrumental findings and has been used to assess constipation in different pathological conditions^14^. In the current study, WexCon was divided into severity subgroups according to cut-off scores as follows: normal (0); mild (1–8); moderate (9–16); severe (> 16).

### Statistical analysis

Statistical analysis was performed using Stata 15.1 (StataCorp, College Station, TX, USA).

For an expected prevalence of 39% (the minimum prevalence estimate reported from previous studies^8^), the required sample size is 458 for an absolute precision of ± 5% in estimating the prevalence with 95% confidence and considering a potential loss of 20%.

Demographic and clinical data are presented as mean ± standard deviation (SD) or median (interquartile range, IQR) as appropriate. Comparisons between groups (RRMS vs PMS) and pwMS with low disability (EDSS < 3.0) vs pwMS with moderate-to-high disability (EDSS ≥ 3.0) were performed using t-test or chi squared test according to the nature of the variables, and Mann–Whitney two-sample statistics if the variable was not normally distributed. Pearson correlation coefficients were assessed between variables of interest. In order to assess the predictive value of NBDS, we fitted a logistic model using as dependent variable presence/absence of bowel impairment by dichotomizing the NBDS score (0 = no impairment, 1 = bowel impairment) and as independent variables the WexInc and WexCon scales as continuous variables.

The Area Under Receiver Operating Characteristic Curve (AUC), sensitivity and specificity were calculated.

The level of statistical significance was set at p < 0.05.

## Results

Four-hundred and forty-seven patients (38% men, 62% women) were recruited in the study. Demographic and clinical features are summarized in Table 1. Two-hundred and ninety-three (65.5%) pwMS were on the following disease modifying treatments (DMTs): teriflunomide (57 pwMS, 12.7%), dimethyl fumarate (56 pwMS, 12.5%), ß-interferon (50 pwMS, 11.2%), fingolimod (36 pwMS, 8%), glatiramer acetate (32 pwMS, 7.1%), ocrelizumab (27 pwSM, 6.2%), natalizumab (17 pwMS, 3.8%), cladribine (4 pwMS, 0.8%), ozanimod (3 pwMS, 0.6%), azathioprine (2 pwMS, 0.4%), siponimod (1 pwMS, 0.2%); data was missing in 7 pwMS and the remaining 154 pwMS were not on DMTs.

**Table 1:** Clinico-demographic data of the MS group as a whole and divided according to disease course.

Variables	MS (n = 447)	RRMS (n = 330)	PMS (n = 117)	PMS vs RRMS
Age (years)	51.1 (11.1)	48.6 (10.4)	57.8 (10.2)	< 0.001
Sex (M/F)	170/277	121/209	49/68	n.s.
Disease duration, years (SD)	21.3 (10.9)	18.7 (9.6)	28.5 (11.1)	< 0.001
EDSS (IQR)	3.0 (2.0–5.0)	2.0 (1.5–3.0)	6.0 (5.5–7.0)	< 0.001
MSSS (SD)	3 (2.3)	2.2 (1.7)	5.3 (2.5)	< 0.001
NBDS (IQR)	2 (0–4)	0 (0–2)	6 (2–9)	< 0.001
WexInc (IQR)	0 (0–1)	0 (0–0)	2 (0–5)	< 0.001
WexCon (IQR)	2 (0–8)	1 (0–2)	10 (2–15)	< 0.001

All values are reported as mean (standard deviation, SD) except for EDSS, NBDS, WexInc and WexCon, reported as median (interquartile range, IQR).

*RRMS* relapsing remitting multiple sclerosis, *PMS* progressive multiple sclerosis, *EDSS* expanded disability status scale, *MSSS* multiple sclerosis severity score, *NBDS* neurogenic bowel dysfunction score, *WexInc* Wexner incontinence scale, *WexCon* Wexner constipation scale.

Frequencies as well as clinico-demographic features for each NBDS subgroup were calculated (Table 2). Similarly, WexCon and WexInc were divided into 4 severity subgroups, from normal to severe, and clinico-demographic features are reported in Table 2.

**Table 2:** Clinico-demographic features of the patients divided into different subgroups according to scores in the different scales.

Subscales (% pts)	Age (years)	Sex (%M)	MS phenotype (%RRMS)	Disease duration (years)	Median EDSS (interquartile range)	Mean MSSS (SD)
NBDS						
Normal (46.3%)	45.2 ± 10.3	46.9	89.9	18.1 ± 10.1	2.0 (1.5–3.0)	1.8 ± 1.5
Very minor (40.3%)	55.2 ± 8.8	25.6	72.8	23.7 ± 11	3.5 (2.5–5.5)	3.2 ± 1.5
Minor (8.3%)	59.6 ± 9.4	40.5	24.3	25.5 ± 10	7.0 (6.5–7.3)	6.8 ± 1.7
Moderate (4.2%)	57.3 ± 11.3	57.9	21.1	23.9 ± 10.4	7.0 (6.5–7.5)	6.9 ± 2.3
Severe (0.9%)	56.8 ± 7.1	25	0	29.2 ± 15.6	7.5 (6.3–8.8)	8.0 ± 2.1
WexInc						
Normal (72.9%)	48.5 ± 10.7	37.8	85.5	19.7 ± 10.3	2.0 (1.5–3.5)	2.2 ± 1.7
Mild (20.6%)	57.8 ± 8.7	35.9	48.9	25.7 ± 11.6	5.8 (3.0–6.5)	4.5 ± 2.4
Moderate (5.4%)	56.6 ± 10.3	41.7	20.8	25.2 ± 11.1	7.0 (6.5–7.5)	6.8 ± 2.0
Severe (1.1%)	67.7 ± 11.3	60	20	22.4 ± 12.7	8.0 (6.5–8.0)	8.1 ± 1.8
WexCon						
Normal (29.1%)	43.2 ± 9.7	49.2	91.5	17.8 ± 10.2	1.5 (1.0–2.5)	1.5 ± 1.2
Mild (49.4%)	52.4 ± 9.8	31.2	82.4	21.3 ± 10.6	3.0 (2.0–4.5)	2.7 ± 1.8
Moderate (16.1%)	58.8 ± 8	36.1	38.9	26.4 ± 10.4	6.0 (4.6–7.0)	5.2 ± 2.1
Severe (5.4%2)	58 ± 12.3	45.8	4.2	25.2 ± 11.7	7.3 (6.5–7.8)	7.4 ± 2.1

*RRMS* relapsing remitting SM, *pts* patients, *NBDS* neurogenic bowel dysfunction score, *WexCon* Wexner Constipation, *WexInc* Wexner Incontinence.

The majority of the patients fell into the “normal” category both for NBDS (46.3%) and WexInc (72.9%), whereas the most represented subgroup with respect to WexCon was the mildly affected (49.4%). In all the scales, RRMS patients presented lower scores than PMS: only 21% of patients belonging to the “moderate” and to the “severe” subgroups of NBDS and WexInc were RRMS.

NBDS, WexInc and WexCon were significantly associated with EDSS, MSSS (all r ≥ 0.61, p < 0.001) even after correcting for age and sex as shown in Table 3. WexInc and WexCon were also correlated with disease duration. Finally, the three bowel dysfunction scores were significantly associated with each other and with disease duration.

**Table 3:** Correlation coefficients between clinical variables and bowel dysfunction scales.

	Disease duration	EDSS	MSSS	NBDS	WexInc	WexCon
NBDS	0.24	0.78	0.74	–	0.84	0.94
WexInc	0.16	0.61	0.61	0.84	–	0.84
WexCon	0.26	0.79	0.75	0.94	0.84	–

*EDSS* expanded disability status scale, *MSSS* multiple sclerosis severity score, *NBDS* neurogenic bowel dysfunction score, *WexInc* Wexner incontinence scale, *WexCon* Wexner constipation scale.

Correlation coefficients are reported, all p values are < 0.001.

Clinical features as well as NBDS, WexInc and WexCon were significantly different when pwMS with low disability (EDSS < 3.0) were compared with pwMS with EDSS ≥ 3.0 (Table 4). In particular, 51.2% of pwMS with low disability presented with bowel symptoms, mainly represented by mild constipation (44%) vs 92.5% patients with EDSS ≥ 3.0 presenting with bowel dysfunctions, usually represented by the simultaneous presence of incontinence and constipation (42.9% of pwMS).

**Table 4:** Clinical features and NBDS, WexInc and WexCon scales in pwMS with low disability compared to pwMS with moderate-to-high disability.

	EDSS < 3.0 n = 209	EDSS ≥ 3.0 n = 238	p
Disease duration, mean (sd)	17.6 (9.6)	24.6 (10.9)	< 0.001
MSSS, mean (sd)	1.33 (1.0)	4.46 (2.2)	< 0.001
NBDS, median (IQR)	0 (0–2)	4 (2–7)	< 0.001
WexInc, median (IQR)	0 (0–0)	0 (0–3)	< 0.001
WexCon, median (IQR)	0 (0–2)	7 (2–11)	< 0.001
No symptoms, n (%)	102 (48.8)	18 (7.5)	< 0.001
Constipation + incontinence, n (%)	10 (4.8)	102 (42.9)	< 0.001
Only incontinence, n (%)	5 (2.4)	5 (2.1)	< 0.001
Only constipation, n (%)	92 (44.0)	113 (47.5)	< 0.001

**Figure 1:**
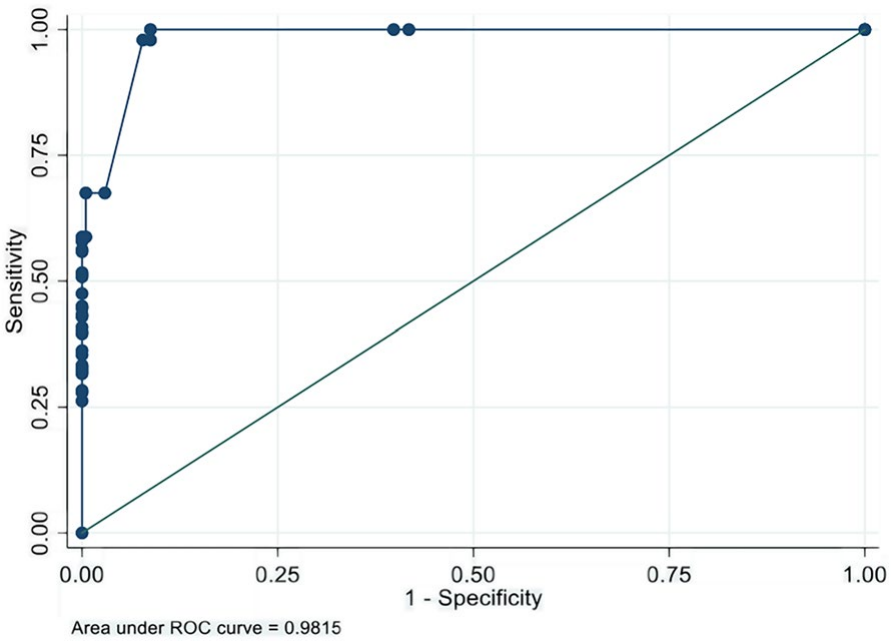
ROC curve for NBDS and Wexner scales as independent variables.

Figure 1 reports the ROC curve obtained after fitting the logistic model: WexInc and WexCon scales can predict nearly perfectly NBDS (AUC = 0.9815). The overall rate of correct classification is estimated to be 94.84, with 91.26% of the pwMS without symptoms correctly classified (specificity) and 97.92% of the pwMS with constipation and incontinence correctly classified (sensitivity).

## Discussion

In the current study, the prevalence of bowel dysfunctions defined as NBDS > 0 in the whole MS cohort was 53.7%, whereas the prevalence of constipation defined as WexCon > 0 was 70.9% and the fecal incontinence prevalence, defined as WexInc was 27.3%.

The attempt to better characterize bowel involvement in MS has underlined some open issues. For example, the prevalence of bowel dysfunctions varies widely among studies, depending on methods used to assess the presence of bowel impairment and on clinical and demographic features of the patients selected for the study^8^. Our monocentric study involving a large sample of consecutively recruited pwMS overcomes some of the limitations of previous studies, related to small sample sizes and retrospective assessment of the symptoms, confirming a high prevalence of bowel impairment in MS. Moreover, bowel dysfunctions are associated with longer disease duration, higher disability and disease severity, as assessed with both EDSS and MSSS. The latter is a powerful tool to assess clinical disability in MS, which was recently demonstrated to considerably improve the individual accuracy of prognostics in MS^10,15^. Accordingly to the aforementioned data, bowel impairment is more frequent in progressive patients. Interestingly, our cohort of patients, despite the long mean disease duration, presented with a relatively low clinical disability as demonstrated by a median EDSS of 3.0, confirming that bowel symptoms can be present regardless of the severity of the disease. To provide further support this finding, we also analyzed pwMS with EDSS < 3.0, detecting mild bowel symptoms also in 268 patients with very low disability, with a mean NBDS 0.8. Indeed, a recent study described bowel symptoms even before the first clinical episode suggestive for MS^16^.

Another strength of our study resides in the use of Wexner scores for incontinence and constipation to separately assess the two main problems related to bowel dysfunctions. The aforementioned scales have been widely applied and validated in different settings, they are quick and easy to use and overcome one of the major limitations of several studies, in that constipation and fecal incontinence were not separately analyzed^6,17^.

In our study, constipation was almost three times more frequent than fecal incontinence. Other studies separately assessing the two symptoms, with a sample size of at least 100 pwMS, reported a prevalence of 18–43% for the former and ranging between 3.4 and 51% for the latter^8^. Both scores were associated with disease duration, clinical disability and disease severity. Furthermore, they were closely associated with each other and with NBDS, which has been repeatedly used in MS^6,17^.

When considering only pwMS with moderate-to-severe constipation (21.5% pwMS) and fecal incontinence (5.5%), they were characterized by high disability and a progressive phenotype, suggesting that sphincter dysfunctions, although present from the beginning and in milder forms, progressively evolve over time and are associated with more severe disease.

As of now, the underlying causes of bowel dysfunction have not been clearly identified. However, prior studies suggest that it is related to spinal cord damage^18,19^ and associated with impaired ambulation^2^. Even though we did not perform a separate analysis with either the EDSS pyramidal subscore or ambulation index, the median EDSS of subgroups of patients characterized by moderate or severe bowel dysfunctions was 7.0, which corresponds to a very limited mobility, depending on the use of a wheelchair to move more than 5 m. Therefore, our findings further support the association of severe bowel dysfunction with MS forms characterized by very limited mobility, as the latter can contribute to both constipation and higher frequency of episodes of fecal incontinence.

A better qualitative and quantitative characterization of the type of symptoms attributable to bowel dysfunctions provided by the current study is not only of scientific interest, but has also relevant potential consequences at a clinical level. The currently available literature evidence on the efficacy of pharmacological and mechanical intervention to treat bowel dysfunctions in MS is moderate at best^20,21^. Very often, limited time during the neurological examination affects the possibility to properly assess sphincter impairment, as reported in a recent study^22^. However, the ability to separately and reliably assess the type and severity of symptoms, with a validated, easy and quick-to-compile scales such as the WexCon and WexCon scales, offers an accessible strategy to further research regarding bowel dysfunction in MS, with potential favorable consequences in terms of treatment management.

Our study is not without limitations. First, the study was carried out in an outpatient setting, possibly limiting the inclusion of severely disabled pwMS. Second, clinical scales were not analyzed with respect to an instrumental bowel assessment that could offer more objective measures of impairment. However, patient-reported outcomes such as the self-administered questionnaires used in our study have been increasingly integrated into clinical and scientific practice, as they offer an easy-to-use method to gather quantitative and qualitative information on different aspects of MS. Third, we did not collect information on symptomatic treatments that could have an impact on the bowel functions and fourth we did not collect data on the sphincter function score of the EDSS, to verify whether there was an association with the scales reported in the study. Finally, we acknowledge that the sex ratio was atypical with respect to most MS studies. Although we did not collect specific reasons for declining to participate, we speculate that it may be the case that women are more likely to be reluctant to discuss problems related to bowel dysfunction.

In conclusion, our study confirms the clinical relevance of bowel dysfunctions in a large group of pwMS, and adds valuable information on the quality and severity of symptoms attributable to bowel impairment. Incorporating the WexCon and WexCon scales into routine clinical practice may facilitate the better monitoring and subsequent management of PwMS.

## Data Availability

The datasets used and/or analyzed during the current study available from the corresponding author on reasonable request. Financial statement: This work was supported by the Italian Ministry of Health “Ricerca Corrente 2022–2024” granted to IRCCS Mondino Foundation.
